# Characterization of wheat straw-degrading anaerobic alkali-tolerant mixed cultures from soda lake sediments by molecular and cultivation techniques

**DOI:** 10.1111/1751-7915.12272

**Published:** 2015-03-04

**Authors:** Katharina Porsch, Balázs Wirth, Erika M Tóth, Florian Schattenberg, Marcell Nikolausz

**Affiliations:** 1 Department of Bioenergy, Helmholtz Centre for Environmental Research – UFZLeipzig, Germany; 2 Department of Environmental Microbiology, Helmholtz Centre for Environmental Research – UFZLeipzig, Germany; 3 Department of Microbiology, Eötvös Loránd UniversityBudapest, Hungary

## Abstract

Alkaline pretreatment has the potential to enhance the anaerobic digestion of lignocellulosic biomass to biogas. However, the elevated pH of the substrate may require alkalitolerant microbial communities for an effective digestion. Three mixed anaerobic lignocellulolytic cultures were enriched from sediments from two soda lakes with wheat straw as substrate under alkaline (pH 9) mesophilic (37°C) and thermophilic (55°C) conditions. The gas production of the three cultures ceased after 4 to 5 weeks, and the produced gas was composed of carbon dioxide and methane. The main liquid intermediates were acetate and propionate. The physiological behavior of the cultures was stable even after several transfers. The enrichment process was also followed by molecular fingerprinting (terminal restriction fragment length polymorphism) of the bacterial 16S rRNA gene and of the *mcrA/mrtA* functional gene for methanogens. The main shift in the microbial community composition occurred between the sediment samples and the first enrichment, whereas the structure was stable in the following transfers. The bacterial communities mainly consisted of Sphingobacteriales, Clostridiales and Spirochaeta, but differed at genus level. *Methanothermobacter* and *Methanosarcina* genera and the order Methanomicrobiales were predominant methanogenes in the obtained cultures. Additionally, single cellulolytic microorganisms were isolated from enrichment cultures and identified as members of the alkaliphilic or alkalitolerant genera*.* The results show that anaerobic alkaline habitats harbor diverse microbial communities, which can degrade lignocellulose effectively and are therefore a potential resource for improving anaerobic digestion.

## Introduction

Lignocellulose-rich wastes have high potential as substrates for biogas production via anaerobic digestion (Kumar *et al*., 2008). Stems, stalks and leaves of crops such as corn, wheat, rice and sugarcane, hulls of grains, woody crops and forest residues etc. are good examples for lignocellulosic waste. In addition, dedicated energy crops (e.g. perennial grasses) and waste products of the paper and forest industry also include lignocellulosic biomass. Despite the energy potential conserved in the polysaccharide structure, the cost-effective utilization of lignocellulose is hampered by its recalcitrant nature. Due to the high lignin content, it is difficult to degrade lignocellulosic biomass by microbes under anoxic conditions (van Wyk, 2001; Hendriks and Zeeman, 2009).

Lignocellulosic biomass is commonly used for bioethanol production, and its recalcitrant structure is broken up by different pretreatment methods (Hamelinck *et al*., 2005). One of the most promising techniques of chemical pretreatments to improve the substrate accessibility is based on the incubation of biomass with liquid ammonia or with highly concentrated bases, such as sodium hydroxide (NaOH) and calcium hydroxide (Ca(OH)_2_) (Hendriks and Zeeman, 2009). A recent study showed that pretreatment with liquid ammonia alters the crystal structure of cellulose, which makes it less recalcitrant to enzymatic hydrolysis (Bellesia *et al*., 2012). Alkaline pretreatment causes dissolution of the lignin but leads to only a minor solubilization of hemicellulose and cellulose (Alvira *et al*., 2010). Saponification of intermolecular ester bounds is also believed to contribute to the swollen state of the biomass, which makes the cellulose fibers more bioavailable for enzymes or degrading microorganisms (Hendriks and Zeeman, 2009). Moreover, alkaline pretreatment has some practical advantages over using acids (Pavlostathis and Gossett, 1985), particularly when biogas production is considered as subsequent process. After acidic pretreatment, part of the biodegradable organics (mainly hemicellulose) remain in the soluble fraction together with the remaining acids, which necessitate neutralization and makes recycling of residual acids difficult (Mosier *et al*., 2005). In case of alkaline pretreatment, the biodegradable fraction remains mainly in the solid phase and only the non-degradable lignin fraction is solubilized. Therefore, the residual alkali can be separated and recycled (Pavlostathis and Gossett, 1985).

Alkaline pretreatment is already established in the bioethanol sector (Hamelinck *et al*., 2005) and has the potential to be used for enhanced biogas production; however, the elevated pH might require neutralization or a microbial community adapted to alkaline conditions. Such communities can be found in different natural alkaline anoxic habitats. Typical alkaline environments are soda lakes (Jones *et al*., 1998), with low or high salt concentrations. Soda lakes with high salt concentration have typically planktonic vegetation, whereas lakes with low salt concentration possess rather perennial vegetation. Stems and leaves of reed (*Phragmytes australis*) and other perennial wetland grasses (e.g. *Typha, Juncus*) contain considerably more lignocellulose than planktonic vegetation, and their tissue is more comparable to the potential lignocellulosic substrates used in biogas plants. Anaerobic digestion of these lignocellulosic plants occurs naturally in the anoxic sediments of the soda lakes and is also reported to be associated with methane production (Oremland *et al*., 1982; Surakasi *et al*., 2007; Antony *et al*., 2012). Therefore, soda lakes with low salt concentration could be proposed as good sources for novel anaerobic lignocellulose-degrading microorganisms, with desirable characteristic to enhance anaerobic digestion of alkaline pretreated lignocellulose-rich biomass in engineered systems.

Therefore, the first aim of the present study was to enrich anaerobic, alkali-tolerant, lignocellulolytic microbial communities that have a potential for application in the biotechnological biogas production from soda lakes with low salt concentration and perennial wetland vegetation. The enrichment process was monitored by physiological and molecular biological characterization of the enrichment cultures. Changes in the community structure were followed during several transfers of the cultures by terminal restriction fragment length polymorphism (T-RFLP). In addition, predominant bacteria were identified based on the 16S rRNA genes, and sequence information was linked to T-RFLP data. Since methane was detected in the gas fraction of all transfers, a survey of methanogenic Archaea was also conducted by the analysis of the *mcrA/mrtA* gene coding a key-enzyme of methanogenesis (alpha subunit of methyl coenzyme M reductase) (Luton *et al*., 2002). The second aim of this study was to isolate and identify cellulose-degrading microorganisms in order to study the microorganisms involved in the anaerobic hydrolysis of lignocellulose under alkaline conditions in future in more detail.

## Results and discussion

### Physiological and molecular biological characterization of the enrichment cultures

In total, six enrichment cultures growing on wheat straw were obtained from the sediment of the two Hungarian soda lakes Szarvas and Velencei. The two well growing cultures S37°C and S55°C were obtained from sediment of Lake Szarvas and incubated at 37°C and 55°C respectively). The third well growing culture V37°C was obtained from sediment of Lake Velencei and incubated at 37°C. All three were transferred several times and were chosen for a more detailed physiological and molecular biological characterization (Table S1).

#### Physiological characterization of the enrichment cultures

**Figure 1:**
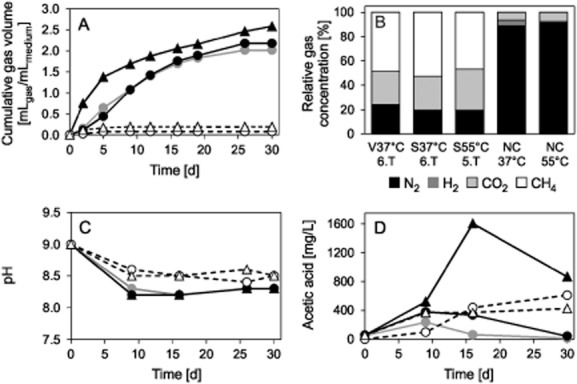
Physiological data of the sixth transfer (6.T) of the enrichment cultures from Lake Velencei (V37°C, 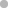) and Lake Szarvas (S37°C, ●) incubated at 37°C and of the fifth transfer (5.T) of the enrichment culture from Lake Szarvas incubated at 55°C (S55°C, ▲) including the negative controls incubated at 37°C (NC37°C, ○) and 55°C (NC55°C, △). The controls contained the same medium and amount of straw as the cultures.A. Normalized semiquantitative cumulative gas production in relation to the medium volume of the cultures and controls.B. Relative gas concentration in the headspace of the cultures and controls after 30 days of incubation.C. pH values.D. Acetic acid concentration in the liquid phase of the cultures and controls.

For each transfer of the three enrichment cultures, the gas production, pH, composition of the gas and liquid phase were followed over time. As example, the data for the sixth transfer of V37°C and S37°C and for the fifth transfer of S55°C (all three cultures were cultivated in parallel) are shown in Fig. 1A–D.

In the first enrichment and in all transfers of the three cultures, the main gas production usually occurred within the first 3 weeks of incubation. In most transfers, the gas production had stopped at the end of incubation (Fig. 1A). The highest gas production occurred during the first enrichment (Fig. S1A) and even after 61 days of incubation, the gas production had not ceased. This can partially be explained by the additional carbon sources introduced with the inoculum itself. In the subsequent transfers, the gas production of the two cultures incubated at 37°C were similar. After 28 to 42 days of incubation between 2.0 and 2.6 mL_gas_/mL_medium_ was produced, except the second and seventh transfer, during which only 0.9–1.3 mL_gas_/mL_medium_ was produced. The gas production of S55°C was with one exception in all transfers higher and lay at the end of incubation between 1.7 and 3.2 mL_gas_/mL_medium_ with the lowest gas production during the second and sixth transfer. The sixth transfer of S55°C and the seventh transfer of S37°C and V37°C were sampled several times to obtain material for the isolation of cellulolytic bacteria, leading to a disturbance of the cultures resulting in lower gas production. The reason for the poor performance of the second transfer is not entirely clear.

Due to the atmosphere of the anaerobic chamber in which the cultivation bottles were prepared, the gas composition in the cultivation bottles consisted of 98% nitrogen (N_2_) and 2% hydrogen (H_2_) at the beginning of the cultivation. During the first enrichment and the following transfers hydrogen was not detected any more in S37°C and V37°C after the first few days of incubation (Table S3). In contrast, traces of hydrogen (< 0.1%) were always detected in the gas phase of S55°C during the whole incubation time. The nitrogen in the culture bottles was replaced over time by carbon dioxide (CO_2_) and methane (CH_4_; Fig. 1B; Table S3). The methane concentration increased constantly over time in all cultures during the first enrichment and transfers. Usually the methane concentration at the end of incubation was similar in the three different cultures and varied between 36% and 58% (Fig. S1C). According to the lower gas production of the second transfer of all three cultures, of the sixth transfer of S55°C and of the seventh transfer of V37°C and S37°C, the methane production in these transfers was also lower than during the other transfers and lay only between 11–34%. During most of the transfers, the carbon dioxide concentration in the headspace of S37°C and S55°C first increased and then decreased. For V37°C, this phenomenon was less pronounced. The carbon dioxide concentration measured in the three cultures during the first enrichment, and the subsequent transfers was in most cases similar and lay at the end of incubation between 25% and 46% (Fig. S1D). The lower gas and methane production of the before mentioned transfers were accompanied by a slightly higher carbon dioxide concentration at the end of the second transfer of all three cultures and of the seventh transfer of S37°C.

The pH was not influenced by the poor performance of the above-mentioned transfers (Fig. S1B). The initial pH of the carbonate buffered medium was 9.0 and dropped in the cultures during incubation (Fig. 1C). At the last day of measurement, the cultures of the first enrichment, as well as the transfers, had a pH of 7.8 to 8.3. An exception was the first transfer, during which the pH of the cultures decreased to pH 7.1–7.5 (Fig. S1B).

In the liquid phase of the cultures, the main compounds produced were acetic and propionic acid. For all the cultures during the first enrichment and during most of the transfers, the acetic acid concentration increased first, and then decreased (Fig. 1D). In the 37°C cultures, this phenomenon was also observed for propionic acid. In contrast, with one exception, the propionic acid concentration of S55°C only increased. With a few exceptions, all cultures produced more acetic acid than propionic acid. The maximum concentration of acetic acid was usually higher in the thermophilic culture (1518–2623 mg l^−1^) than in the two 37°C cultures (847–2143 mg l^−1^). However in a few transfers of the three cultures, the maximum acetic acid concentration was below 400 mg l^−1^. Compared with the acetic acid, the maximum propionic acid concentration was smaller in S55°C (131–220 mg l^−1^) than in the two 37°C cultures (116–544 mg l^−1^). However, due to the sampling interval (approximately once per week), the maximum concentrations of both acids reached in the cultures during the first enrichment, and the transfers might have been higher. From the fourth transfer on another high-performance liquid chromatography (HPLC) system was used and glucose was detected in low concentration (< 50 mg l^−1^) during the transfers of all three cultures. All other substances measured were either not detected (lactic acid in S37°C and V37°C; ethanol in S37°C; n-valeric acid in S55°C) or were only detected from time to time with concentration usually below 50 mg l^−1^ during the first enrichment and the transfers.

Although it was not an intention to enrich methanogenic archaea, the complete biogas process was established and maintained in our cultures, which was indicated by the formation and consumption of acetic acid, coupled to the production of carbon dioxide and methane. The biogas process occurs in natural and artificial systems under anoxic conditions in the absence of an electron acceptor other than carbon dioxide and can be divided in the four steps: hydrolysis, acidogenesis, acetogenesis and methanogenesis (Schink, 1997; Weiland, 2010). Methanogenesis was presumed in the original sediment samples due to anoxic conditions and gas bubbles observed during the sampling. Since the cultures were grown in batch systems, the four steps of the biogas process were temporally shifted. This led to the increase and subsequent decrease of acetate in all three cultures and of carbon dioxide in the cultures from Lake Szarvas. This also explains the increasing methane concentrations in all cultures over time. The formation of propionate in significant amounts suggests that part of the acidogenesis was performed by the propionic acid fermentation (Altschul *et al*., 1997).

According to the gas production, pH, produced gaseous and liquid metabolites the performance of the three cultures was stable and no loss in activity was observed via the eight and nine transfers of the cultures respectively (Fig. S1). Even after the two before-mentioned transfers, during which the activity of the cultures were lower (the second transfer of all three transfers, the sixth transfer of S55°C and the seventh transfer of S37°C and V37°C), the cultures recovered, suggesting no major changes in the community. The stability of the community structure in all transfers was also shown by the molecular biological analysis (see *Community structure of the bacteria*).

The controls had the same composition as the cultures except that they were not inoculated. The controls of the first enrichment showed high gas production (1.2 mL_gas_/mL_medium_), hydrogen production in case of the 37°C control (19% H_2_) and complete hydrogen consumption in case of the 55°C control, high carbon dioxide production (39% at 37°C and 54% at 55°C) and high acetic acid concentrations (1642 mg l^−1^ at 37°C and 2740 mg l^−1^ at 55°C), but no methane formation. These results indicated that one autoclaving step was not enough to kill all the indigenous microorganisms or their spores attached to the straw. However, the microbial community of the controls produced different T-RFLP patterns than the communities of the cultures (see *Community structure of the bacteria*) showing that under the alkaliphilic conditions the microorganisms from the soda lakes dominated. From the third transfer on, the straw was autoclaved twice, and the measured parameters did not indicate microbial growth in the controls of this and the following transfers (see below). Stronger sterilization techniques were not applied in order to avoid a too strong change of the wheat straw structure.

In the controls of the transfers, ≤ 0.3 mL_gas_/mL_medium_ was produced within the first days of incubation due to the warming of the bottles after transferring them to the incubator (Fig. 1A). At the end of incubation, the atmosphere of the controls contained 1–5% H_2,_ which originated mainly from the atmosphere of the anaerobic chamber in which the bottles were prepared (Fig. 1B; Table S3). Additionally, in the 37°C controls, usually 2–6% CO_2_, and in the 55°C controls, 4–11% CO_2_ was present, probably due to the out-gassing of the carbonate buffer which was higher at thermophilic conditions. Methane was never detected in any of the controls. The pH of the controls decreased to a smaller extent than in the cultures, and at the end of incubation, it lay between pH 8.0 and 8.6 (Fig. 1C). In the controls, acetic acid was always detected in increasing concentration with a maximum of 181–1181 mg l^−1^ (Fig. 1D). After changing the HPLC system (see above) also glucose was always detected in the controls, usually with a concentration below 50 mg l^−1^. In the 37°C controls, formic acid was additionally constantly present with concentrations up to 260 mg l^−1^, while in the 55°C control, propionic acid was usually detected in low concentrations (< 50 mg l^−1^). With very few exceptions iso- and n-butyric acid, as well as iso- and n-valeric acid were never present in this control. Since there was no gas production and the gas composition was relatively stable in the controls, the substances detected by HPLC were probably leached out from the straw.

#### Community structure of the bacteria

**Figure 2:**
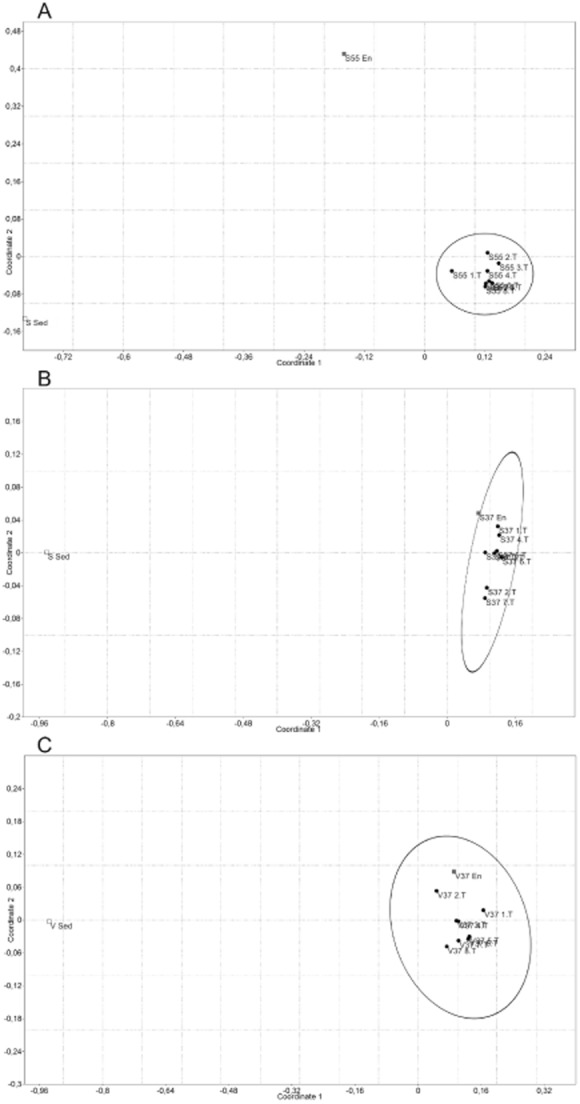
n-MDS plot based on Bray–Curtis dissimilarity index showing the similarity relationships of the microbial communities in enrichment steps obtained from (A and B) the Lake Szarvas (denoted with S) and from (C) Lake Velencei (denoted with V). The analysis is based on the T-RFLP data obtained by MspI restriction enzyme. The ellipses show the 95% confidence interval. Sed – original sample; En – first enrichment step; 1–9.T – further transfer steps; 37 and 55 – incubation temperature [°C].

The bacterial community carries out the first three steps of the biogas process (hydrolysis, acidogenesis and acetogenesis), whereas Archaea are responsible for the last step, the methanogenesis (Weiland, 2010). The bacterial community of the sediment samples, the initial enrichment and the following transfers of the cultures V37°C, S37°C and S55°C were characterized by T-RFLP. In comparison to the sediment samples, the community structure had been significantly changed during the first enrichment step of all three cultures. This is not surprising since the cultivation medium and temperature did not correspond to the conditions in the lake sediments. Based on the statistical analysis of the dissimilarity values between the T-RFLP profiles of the samples, the non-metric multidimensional scaling (n-MDS) ordination analysis grouped the transfers together, while the original sediment samples and the first enrichment step in case of the thermophilic enrichment were outliers (Fig. 2A–C). In contrast, the first enrichment step has already resulted in a similar community structure to the further transfers at 37°C. These results supports the physiological data by showing that transferring the cultures several times have not influenced the microbial community (and their activity) to a great extent. Deoxyribonucleic acid (DNA) was also isolated from few of the controls at 37°C and at 55°C. The DNA yield was negligible, and polymerase chain reaction (PCR) amplification was difficult. In that case, DNA probably originated from dead cells or microbes growing from spores survived the autoclaving. The T-RFLP patterns of the controls were not similar to those obtained from the transfers (data not shown).

It is not surprising that the enrichment process dramatically changed the original community structure and that the predominant operational taxonomic units (OTUs) in the stable enrichment cultures mainly aroused from the background minority of the original sample. The pioneers of the enrichment culture techniques as well as recent studies already implied that the latent microbial life can be resuscitated by providing the appropriate environmental conditions in the laboratory (Baas Becking, 1934; de Wit and Bouvier, 2006). Jacquiod and colleagues (2013) investigated the response of soil bacterial community to chitin enrichment in a microcosm experiments using a metagenomic approach coupled to phylochips and high-throughput shotgun pyrosequencing. A hierarchical classification of metagenomes showed a strong ‘microcosm effect’ as untreated control soil samples clearly separated from the microcosm incubation cluster. Vorob’ev and Dedysh (2008) applied the enrichment culture technique to investigate the structure of methanotrophic communities in peat soil, and significant changes were observed due to cultivation by applying fluorescent *in situ* hybridization technique. However, we have to note that in our study, the inoculation source still had a major influence on the performance of the enrichment cultures. Our attempt to establish a stable, well-performing thermophilic culture was successful using sediment from Lake Szarvas with a thermal inflow, despite the non-thermophilic conditions during sampling. In contrast, we failed to establish such a community from the sediment from Lake Velencei, which is lacking any thermal inflow (data not shown).

**Figure 3:**
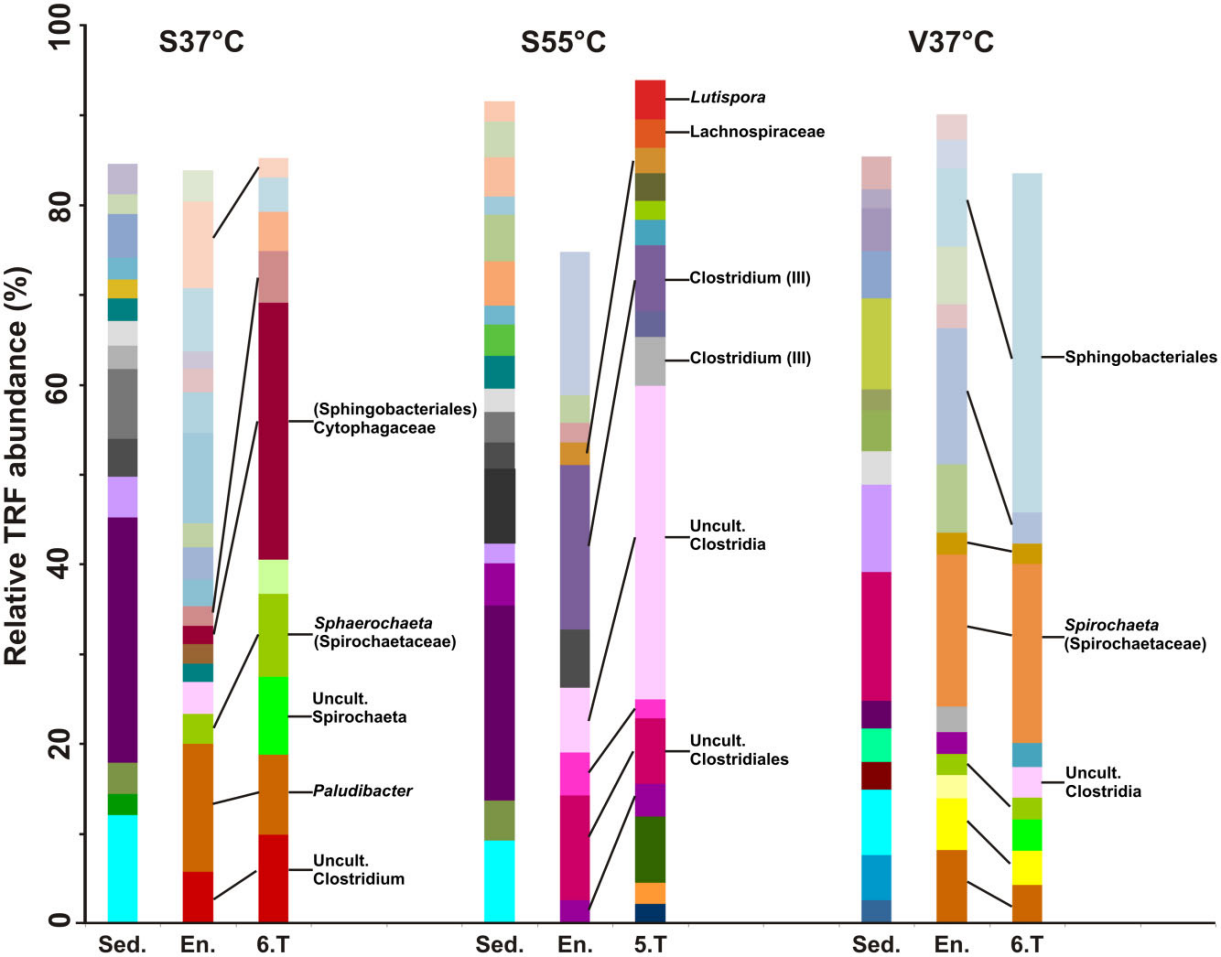
Relative T-RF abundance of the bacterial 16S rRNA genes digested with the restriction enzyme MspI of the sediment samples (Sed), first enrichment culture (En) and sixth or fifth transfer (6.T or 5.T) of the mixed cultures obtained from the Lake Szarvas (S37°C and S55°C) and Lake Velencei (V37°C) incubated at 37°C or 55°C. Terminal restriction fragments with an abundance < 2% are not shown. Each color represents one T-RF and major T-RFs of the same length are connected by lines. Microorganisms producing certain T-RFs were identified by cloning and sequencing.

In order to gain more details about the taxonomy of the community members, clone libraries have been established from the sixth transfer of V37°C and S37°C and from the fifth transfer of S55°C. After screening of the PCR amplicons from the clones by T-RFLP, members of the predominant T-RF groups have been partially sequenced. In this way, we linked taxonomic affiliation to the most dominant T-RF peaks (Fig. 3). The two communities obtained from different lakes under the same condition (incubation temperature 37°C) were completely different at species level (using less than 97% similarity value as definition of distinction). However, looking at the affiliation of the sequences at higher taxonomical ranks, the two cultures can be considered as similar. Both enrichment cultures can be described by the predominance of bacteria affiliated to the orders Sphingobacteriales, Clostridiales and the family Spirochaetaceae.

This may explain the great similarity of the mesophilic cultures regarding the physiological parameters. The main difference between the two cultures was the carbon dioxide concentration. In S37°C, it usually increased and then decreased, whereas in the transfers of V37°C, the carbon dioxide concentration either decreased to a smaller extent or did not decrease at all. The enrichment culture established at 55°C from the sediment taken from Lake Szarvas was predominated by bacteria related to thermophilic members of the genus *Clostridium.* The physiological data reflects this difference in the community structure between the mesophilic cultures and the thermophilic culture. S55°C had a higher gas production, higher acetic acid and lower propionic acid concentration than the 37°C cultures. Additionally, propionic acid usually only accumulated in this culture, but was not consumed during the incubation.

In general, most of the sequences of the three cultures were only distantly related to any cultivable microorganism (Table S4), which highlights the unknown diversity of these lake sediments and the enrichment cultures obtained from them. In general, we can conclude that the degradation of lignocellulose-rich straw is carried out by a diverse microbial consortium, which differs significantly from the original microbial community in the lake sediments.

#### Community structure of the methanogens

**Figure 4:**
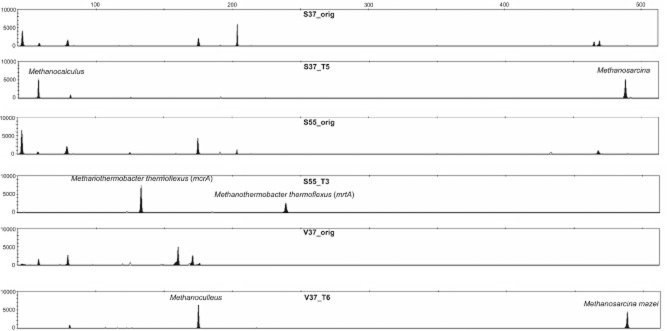
Examples of mcrA gene PCR product T-RFLP patterns showing differences of the methanogenic community structure of the original lake sediment samples and the enrichment cultures. Phylogenetic affiliation of the TRF peaks was achieved by sequence analysis of supporting clone libraries from isolated DNA samples of the same representative samples shown in the figure.

Due to the observation of the stable methane production by the three cultures during the first enrichment and all transfers (see above), the community structures of the methanogenic Archaea were also investigated by *mcrA/mrtA* gene-based molecular approach. The methanogenic community in the sediment samples was relatively divers (6–7 major peaks; Fig. 4), while the diversity decreased during the initial enrichment and further transfers. The most drastic diversity reduction was observed in the S55°C culture where only a single predominant methanogenic microorganism identified as *Methanothermobacter thermoflexus* remained. Two major peaks in the T-RFLP pattern and two sequence types in the clone library of this enrichment culture most probably represented the *mcrA* and the *mrtA* gene of the same microorganism (Fig. S2). Cultures incubated at 37°C were slightly more diverse with two major groups of methanogens and some small peaks representing a minor part of the methanogenic community (taxonomic affiliation was not possible due to the size of the clone library). In both mesophilic enrichment cultures (S37°C and V37°C), one of the two predominant sequence types were affiliated to the genus *Methanosarcina*. The sequence from V37°C represented *Methanosarcina mazei*, whereas the sequence from S37°C was more related to *Methanosarcina thermophila* (Fig. S2). The other predominant sequences in both mesophilic cultures represented members of the order Methanomicrobiales. While the sequence from the V37°C enrichment was affiliated to the genus *Methanoculleus*, sequences from S37°C were more related to a *Methanocalculus* strain.

The *Methanosarcina* spp. are known to have more flexible metabolism, and members of this genus can use aceticlastic, hydrogenotrophic and methylotrophic pathways for methane production. In contrast, the order Methanomicrobiales and the genus *Methanothermobacter* are known as containing only strict hydrogenotrophic methanogens (Demirel and Scherer, 2008). Although sequences affiliated to the strict aceticlastic genus *Methanosaeta* and of the genus *Methanobacterium* were detected in the sediment from the Lake Szarvas, these sequence types were not detected further in the enrichment cultures (Fig. S2). It can be concluded that the thermophilic culture was strict hydrogenotrophic, while the contribution of aceticlastic pathway is presumed in the mesophilic cultures. However, like in the 37°C cultures, the acetic acid concentration in the thermophilc culture first increased and then decreased during incubation (Fig. 1D). Hence, the lack of aceticlastic methane production in the thermophilic culture suggests the prevailing of syntrophic acetate oxidation pathway for acetate conversion in these transfers. Three of the four isolated syntrophic acetate-oxidizing bacteria described so far belong to the Firmicutes-Clostridia class (Muller *et al*., 2013). The great abundance of the sequences affiliated to the order Clostridiales in our thermophilic culture may also hint syntrophic acetate oxidation. The stable conditions during cultivation further narrowed the diversity of the methanogens to few or a single predominant species. Despite this very low methanogenic diversity, the methanogenesis was stable during the investigated period in many consecutive transfers (Fig. S1).

### Isolation of cellulolytic bacteria

Several attempts were made during this study to isolate important cellulolytic members of the enrichment cultures as pure culture to investigate their physiological role in degradation of lignocellulosic substrates, such as straw, in the future. Overall, 106 colonies obtained on agar plates were screened for purity and similarity by T-RFLP. Isolation under thermophilic condition was hampered by slow growth on the agar plate, difficulties in DNA isolation with the simplified protocol and by obtaining pure cultures. Strains were screened for purity by T-RFLP, and grouped into T-RF groups. Selected members of the same T-RF groups were partially sequenced to cover almost the full 16S rRNA gene. The detailed results of sequence analysis to help the taxonomic affiliation of the strains are shown in Table 1. Some of the isolated strains were very closely related (97% similarity or higher) to microorganisms previously isolated from anoxic alkaline environments, such as *Actinotalea fermentans* (Yi *et al*., 2007), *Alkaliflexus imshenetskii* (Zhilina *et al*., 2004), *Natronincola ferrireducens* (Zhilina *et al*., 2009), *Anaerovirgula multivorans* (Pikuta *et al*., 2006) and *Anaerobranca gottschalkii* (Prowe and Antranikian, 2001). In many cases, the taxonomic affiliation was possible only at genus level using 95% similarity threshold (*Clostridium, Alkaliphilus, Sphaerochaeta*). In case of many strains, the sequence similarity values were so low that they probably represent not just new species but members of novel genera. The polyphasic description of these novel strains will be done in the future.

**Table 1:** Sequencing results and taxonomic affiliation of the isolated pure cultures including experimentally determined T-RFs. Temperature and pH optimum, as well as cellulose degradation capability of the closest cultivable relatives are also listed

Isolate [sequence length (bp)]	Acc. no.	HaeIII T-RF [bp]	MspI T-RF [bp]	Highest blast hit	Closest cultivable relative		
				(Acc. no.)/sequence identity	(Acc. no.)/sequence identity	pH/temp. optimum	Cellulose degradation
S37_01_1 (1403)	LK391546	270	221	Uncultured bacterium clone SYNH02_C3-03B-079 (JQ245533)/99%	*Saccharofermentans acetigenes* (AY949857)/91%	6.5/37°C	−
S37_03_1 (1428)	LK391549	270	221	Uncultured bacterium clone SYNH02_C3-03B-079 (JQ245533)/99%	*Saccharofermentans acetigenes* (AY949857)/91%	6.5/37°C	−
S37_24_1 (1416)	LK391559	79	66	Uncultured bacterium clone: HDBW-WB62 (AB237725)/96%	*Clostridium* sp. AN-AS6C (FR872932)/92%	n.a.	n.a.
S37_11_2 (1412)	LK391555	79	66	Uncultured bacterium clone: HDBW-WB62 (AB237725)/96%	*Clostridium* sp. AN-AS6C (FR872932)/92%	n.a.	n.a.
V37_10_1 (1012)	LK391571	237	295	*Clostridium phytofermentans* ISDg (CP000885 )/96%	*Clostridium phytofermentans* ISDg (CP000885 )/96%	6.0–9.0/37°C	+
V37_02_2 (1019)	LK391564	237	295	*Clostridium phytofermentans* ISDg (CP000885 )/96%	*Clostridium phytofermentans* ISDg (CP000885 )/96%	6.0–9.0/37°C	+
V37_06_2 (858)	LK391569	237	295	*Clostridium phytofermentans* ISDg (CP000885 )/96%	*Clostridium phytofermentans* ISDg (CP000885 )/96%	6.0–9.0/37°C	+
S37_06_2 (1023)	LK391550	238	295	Uncultured bacterium clone G35_D8_H_B_G07 (EF559175)/96%	*Clostridiaceae* bacterium FH052 (AB298768)/95%	n.a.	n.a.
S37_08_2 (637)	LK391552	238	295	Uncultured compost bacterium clone BO2441 (FN667328)/96%	*Clostridiaceae* bacterium FH052 (AB298768)/93%	n.a.	n.a.
S37_02_2 (1395)	LK391548	290	460	Uncultured bacterium clone: MPB1-156 (AB630538)/91%	*Clostridiaceae* bacterium SH032 (AB377176)/91%	n.a.	n.a.
S37_09_2 (1399)	LK391554	281	278	Uncultured bacterium clone SV79-4 (JQ316685)/94%	*Cellulosilyticum lentocellum* DSM 5427 (CP002582 )/94%	7.5–7.7/40°C	+
S37_30_1 (1392)	LK391560	281	278	Uncultured bacterium clone NT4_C114 (HM630230)/94%	*Cellulosilyticum lentocellum* DSM 5427 (CP002582 )/94%	7.5–7.7/40°C	+
S37_07_1 (1394)	LK391551	227	129	*Actinotalea fermentans* (AB639014)/99%	*Actinotalea fermentans* (AB639014)/99%	7.4/30–37°C	+
S37_01_2 (1359)	LK391547	227	128	*Actinotalea fermentans* (AB639014)/99%	*Actinotalea fermentans* (AB639014)/99%	7.4/30–37°C	+
S37_13_2 (1294)	LK391557	31	462	Uncultured bacterium clone mesophilic_alkaline-57 (GU455172)/98%	*Alkaliflexus imshenetskii* strain DSM 15055T (FR839023)/97%	8.5/35°C	−
V37_03_2 (1327)	LK391566	31	462	Uncultured bacterium clone mesophilic_alkaline-57 (GU455172)/98%	*Alkaliflexus imshenetskii* strain DSM 15055T (FR839023)/99%	8.5/35°C	−
V37_05_2 (1325)	LK391568	31	462	*Alkaliflexus imshenetskii* type strain DSM 15055T, isolate Z-7010 (FR839023)/99%	*Alkaliflexus imshenetskii* strain DSM 15055T (FR839023)/99%	8.5/35°C	−
S37_18_1 (1420)	LK391558	235	293	*Natronincola ferrireducens* strain Z-0511 (EU878275)/100%	*Natronincola ferrireducens* strain Z-0511 (EU878275)/100%	8.4–8.8/35–37°C	−
V37_02_1 (1421)	LK391563	236	311	*Anaerovirgula multivorans* strain SCA (NR_041291)/96%	*Anaerovirgula multivorans* strain SCA (NR_041291)/96%	8.5/35°C	+
V37_05_1 (1401)	LK391567	236	311	*Anaerovirgula multivorans* strain SCA (NR_041291)/96%	*Anaerovirgula multivorans* strain SCA (NR_041291)/96%	8.5/35°C	+
V37_09_2 (1012)	LK391570	236	311	Bacterium enrichment culture clone T12RRH100B1 (HQ896297)/98%	*Anaerovirgula multivorans* strain SCA (NR_041291)/97%	8.5/35°C	+
S55_27_1 (1458)	LK391562	300	550	*Anaerovirgula multivorans* strain SCA (NR_041291)/100%	*Anaerovirgula multivorans* strain SCA (NR_041291)/100%	8.5/35°C	+
V37_03_1 (1420)	LK391565	235	293	Uncultured bacterium clone WF16S_44 (EU939394)/98%	*Clostridiaceae* bacterium E2R (EU627628)/96%	n.a.	n.a.
S37_09_1 (1390)	LK391553	198	149	Uncultured bacterium clone 23 (JX898065)/95%	*Turicibacter sanguinis* strain MOL361 (NR_028816)/95%	7.5/37°C	n.a.
S55_26_2 (1402)	LK391561	229	170	*Anaerobranca gottschalkii* strain LBS3 (NR_025050)/97%	*Anaerobranca gottschalkii* strain LBS3 (NR_025050)/99%	9.5/50–55°C	+
S37_12_1 (1138)	LK391556	62	123	Uncultured bacterium clone Bac_SB_52 (JQ739121)/99%	*Spirochaeta* sp. Buddy (CP002541)/99%	n.a.	n.a.

Acc. no., accession number; bp, base pair; n.a., information is not available.

However, based on the T-RFLP patterns of the colonies and the cultures, the isolation strategy on agar plates missed the most abundant members and perhaps the key players of the liquid cultures. Such difference between the liquid cultivation and the isolation on agar plate was not expected, because the same liquid medium was used for the preparation of the plates. The utilization of cellobiose as a new single or supplemented carbon source in addition to the complex substrate wheat straw might be partially responsible for this result. Based on the literature, it can be assumed that cellulose degraders can utilize cellodextrins including cellobiose, as a great number of the investigated cellulose-degrading strains possess intracellular cellobiose phosphorylases (Lynd *et al*., 2002). Cellobiose dissolves well in the cultivation medium, whereas the homogenous distribution of the insoluble cellulose and wheat straw was not perfect. Therefore, they were not that appropriate carbon sources for obtaining single colonies on the surface of the agar plates. Cellobiose has been chosen over glucose because a better utilization was described by well-known anaerobic cellulose degraders (e.g. Thurston *et al*., 1993). However, in complex communities non-cellulolytic species can compete for cellodextrin products (cellobiose) of cellulose hydrolysis, but they may also play an important regulatory role in consumption of these hydrolysis products (Maglione *et al*., 1997).

In case of isolation attempt with cellobiose (as a single carbon source or as a supplemented major carbon source), these associated non-cellulolytic species or cellulolytic but non-adherent bacteria have perhaps selective growth advantage. We have to note that most of the closest relatives of our cultures are reported to be capable of cellulose utilization (Table 1). We may also assume that cellulose degradation of the predominant members of the liquid culture requires syntrophic interactions. Many cultures were discarded after the T-RFLP screening as not pure, containing more than one microorganism. Some of these ‘mixed’ T-RFLP patterns containing the same double peaks might indicate non-methanogenic syntrophic interactions (data not shown).

The study demonstrated that sediments of soda lakes are excellent sources of novel lignocellulose-degrading microorganisms tolerating alkaline conditions. The anaerobic degradation of wheat straw coupled to methanogenesis by three stable complex microbial consortia was demonstrated during several transfers. Effective methanogenesis was maintained by one or few predominant species, while lignocellulose hydrolysis and fermentation of sugars were done by a complex bacterial community. Although the attempt to isolate lignocellulolytic microorganisms resulted in many novel strains, we failed to isolate the most abundant members of the liquid enrichment cultures. Improved isolation strategies are needed in the future to get these important microorganisms in pure culture.

## Experimental procedures

### Sampling

Sediment samples were collected from two Hungarian soda lakes, the Lake Velencei (47°14’2.81”N; 18°39’2.90”E) and the Lake Therm-Organ (situated close to Szarvas, therefore denoted as Lake Szarvas in the text) (46°554’07.31”N; 20°36’19.78”E), in October 2011. Lake Velencei is a shallow soda lake with an average depth of 1.4 m and a surface area of 25 km^2^. Three sediment samples were taken from 1.5 m depth. On the sampling day, the water body had the following main characteristics: Temperature 19.5°C; pH 9.02; conductivity 3.21 mS cm^−1^ and 9.04 mg l^−1^ dissolved oxygen. The Lake Szarvas is a small reservoir formed from an oxbow of the Hármas-Körös River. It is used to store exhausted water which is collected from thermal springs and is first used for heating buildings and greenhouses nearby. This thermal inflow (800–1000 l min^−1^, 50–70°C) is also responsible for phenol contamination of 1–3 mg l^−1^ in the lake. Three sediment samples were taken from 0.5 m depth. On the sampling day, the water body had the following main characteristics: temperature 21.0°C; pH 9.03; conductivity 6.28 mS cm^−1^ and no dissolved oxygen was detected 2 cm below the surface. Sediment samples were taken with 100 ml glass bottles around the reed bed and also further away from the vegetation. The bottles were completely filled with sediment and water, closed with airtight lids and put into anoxic atmosphere generation bags Anaerocult A mini (Merk Millipore, Germany). The samples were stored at room temperature until further processing in the laboratory.

### Enrichment and cultivation

The modified DSMZ medium 1036 was used for the first enrichment and subsequent cultivation. The medium consisted of 0.5 g NH_4_Cl, 0.2 g KH_2_PO_4_, 0.1 g MgCl_2_ × 6H_2_O, 0.2 g KCl, 2.0 g NaCl, 0.2 g yeast extract, 1.0 ml trace element solution SL10 (DSMZ medium 320) and 0.5 mg resazurin dissolved in 850 ml high-purity water. The medium was stirred for at least 30 min in an anaerobic chamber (98% N_2_, 2% H_2_) to make it anoxic. After autoclaving it (20 min, 121°C, 1 bar), following heat labile, anoxic and sterile solutions were added (values in brackets are the concentration of the stock solution): 12 ml cysteine-HCl monohydrate (30 g l^−1^), 34 ml Na_2_CO_3_ (29.41 g l^−1^), 100 ml NaHCO_3_ (76 g l^−1^), 4 ml selenite-tungstate (DSMZ medium 385, 1:4 diluted with high-purity water). Wheat straw milled to 10 mm length served as the lignocellulose source and consisted of 445 ± 31 g cellulose/kg_TS_, 336 ± 3 g hemicellulose/kg_TS_ and 72 ± 3 g lignin/kg_TS_ [values are averages of three measurements and were determined according to the standard procedure (Van Soest *et al*., 1991; Naumann and Bassler, 2006)].

For the enrichment and cultivation 50, 100 or 250 ml glass bottles were used. First, the bottles were only filled with straw (0.5 g straw/50 ml medium) and 1 ml high-purity water, autoclaved (30 min, 121°C, 1 bar) and stored overnight in an anaerobic chamber. In the anaerobic chamber, high-purity water (1 ml) was added again, the bottles were closed with butyl rubber stoppers and autoclaved a second time (30 min, 121°C, 1 bar). The second autoclaving step was introduced from the third transfer on. Second, the medium was added anaerobically and sterile to the bottles after autoclaving (25 ml medium into 50 ml-bottles, 50 ml into 100 ml-bottles and 100 ml into 250 ml bottles).

Enrichment cultures from each of the three different sediment samples of each lake were setup. For the first enrichment, the bottles containing medium and straw were opened in the anaerobic chamber, 2.1 g to 5.7 g of sediment was added, and the bottles were closed again. For further cultivation, fresh medium and straw was inoculated from the previous transfer to obtain about 2 × 10^6^ cells ml^−1^. The cell concentration of the previous transfer was determined microscopically. From the three cultures per lake, two were incubated at 37°C and one at 55°C to enrich mesophilic as well as thermophilic lignocellulolytic microorganisms (Table S1). Negative controls were prepared in the same way as the bottles for the cultures except that they were not inoculated.

### Isolation of cellulolytic microorganisms

From the seventh and eighth transfer of three selected enrichment cultures, namely S37°C and S55°C (both cultures were enriched from sediment from the Lake Szarvas and incubated at 37°C and 55°C, respectively) and V37°C (enriched from sediment from the Lake Velencei and incubated at 37°C), cellulolytic microorganisms were isolated on agar plates. The plates were prepared with the medium described above and contained additionally 1.5% (m/v) agar and five different cellulose sources: (i) 1% (m/v) 0.5 mm milled wheat straw, (ii) 0.5% (m/v) crystalline cellulose, (iii) 0.2% (m/v) cellobiose, (iv) mix of 1% (m/v) 0.5 mm milled wheat straw and 0.5% (m/v) crystalline cellulose and (v) mix of 1% (m/v) 0.5 mm milled wheat straw and 0.2% (m/v) cellobiose. Wheat straw and 1 ml of high-purity water were autoclaved (30 min, 121°C, 1 bar) in a glass bottle and stored overnight in the anaerobic chamber. After adding medium and agar and in cases of mixed carbon sources, also cellulose or cellobiose, the bottle was incubated for 2 h at 55°C and subsequently autoclaved (30 min, 121°C, 1 bar). For medium containing only cellulose or cellobiose, the carbon source was added to the medium with the agar directly before autoclaving (20 min, 121°C, 1 bar) without any pre-incubation. The heat labile substances (see above) were added to 60°C warm medium before the plates were poured in the anaerobic chamber. Although the crystalline cellulose did not dissolve, it was more or less homogenously distributed in the agar. Since the straw settled to the bottom of the agar plates, plates containing only wheat straw were turned around in the Petri dish after solidification to enhance the contact possibilities between plated microorganisms and straw particles. Plates containing a mixed substrate were not turned around, since in these set-ups, the straw served only as source for additional nutrients leached out from the straw particles.

The selected enrichment cultures were 10-fold diluted in series until the factor 10^−6^ in the described medium with additives but without (ligno)cellulose source. From each dilution, 100 μL were plated on the agar plates in the anaerobic chamber, and the plates were anaerobically incubated at 37°C (S37°C, V37°C, S55°C) and 55°C (S55°C). In the anaerobic chamber, 15 to 30 colonies from each culture were picked with sterile tooth picks and transferred to fresh agar plates. Colonies from plates with only one substrate were transferred with two exceptions to plates with cellobiose and colonies from plates with mixed substrates, and the two exceptions were transferred to plates with straw–cellobiose mix. The incubation conditions of the plates with transferred colonies were the same as described above. Colonies growing on these plates were analysed using molecular biological techniques described below.

### Physiological characterization of liquid cultures

#### Gas volume

The gas volume was measured semi-quantitatively with a horizontal U-tube (Fig. S3). The tube was filled with a solution of 200 g l^−1^ NaCl and 5 g l^−1^ citric acids which was displaced by the gas inflow (Rozzi and Remigi, 2004). The culture bottles were connected to the U-tube by a syringe with needle, and the measured gas volume was normalized to standard pressure (101.325 kPa) and temperature (273.15 K) with following equation:

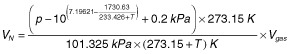


With V_N_ being the normalized gas volume, *p* the ambient pressure [kPa], *T* the ambient temperature [°C] and V_gas_ the measured gas volume. The measurement of the gas volume led to a depressurization of the bottles, and all further sampling of the bottles were performed after this procedure.

#### Gas composition

The headspace of the bottles was sampled with a 1 ml syringe previously flushed with nitrogen, and a 1 ml gas sample was immediately transferred into a 20 ml glass vial filled with argon. The gas composition was analysed with the gas-phase chromatograph Micro GC CP-2002P (Chrompack, the Netherlands) containing two parallel columns, the column Molsieve 5Å PLOT (30 m length, 0.53 mm diameter, 25 μm film) separating H_2_, N_2_ and CH_4_ at 30°C, and the column HayeSep A (15 m length, 0.53 mm diameter, 20 μm film) separating CO_2_ at 50°C. The glass vial with the sample was connected to the gas phase (GC). After the GC program was started, the sample was injected for 5 ms. Argon was used as carrier gas. The gases were detected with a thermal conductivity detector and analysed with the software Maestro (Chrompack). The detector modus of the Molsieve column was set to low sensitivity, that of the HayeSep column to high sensitivity. The gases H_2_, N_2_, CO_2_ and CH_4_ were normalized to 100%.

#### Organic acids, ethanol, glucose, pH

Until the third transfer, the liquid samples were analysed as follows: With an anoxic syringe, 900 μl of the liquid phase were sampled, centrifuged (10 min, 20817 *g*, 4°C), and the supernatant was stored at −20°C. At the day of measurement, the samples were thawed, mixed, filtered (0.2 μm, regenerated cellulose) and the filtrate was used for HPLC. The samples were stored at 4°C in the autosampler until measuring. The chromatograph (Shimadzu, USA) had a Nucleogel Ion 300 OA column (300 mm length, 7.8 mm diameter, Macherey-Nagel, Germany). The oven temperature was set to 70°C, the injection volume to 20 μl. Five micromolar sulfuric acid (H_2_SO_4_) was used as eluent with a flow rate of 0.6 ml min^−1^. The running time of a sample was 40 min. Following substances were detected with a reflexion index detector and analysed with the software Class-VP (Shimadzu, Japan): glucose, ethanol, lactic acid, formic acid, acetic acid, propionic acid, iso-butyric acid, n-butyric acid, iso-valeric acid and n-valeric acid. In order to prevent an overload of the columns, most of the samples were diluted with high-purity water. Hence, substances in low concentrations could not be detected. Additionally, the retention time of n-butyric acid and ethanol were very similar, and a complete analytic separation was not possible. From the fourth transfer on, another HPLC system was used, which required modified sample preparation. A liquid sample of 900 μl was taken from the cultures with an anoxic syringe, centrifuged (10 min, 20817 *g*, room temperature), and the supernatant was stored at −20°C. For the analysis, the sample was thawed, mixed, centrifuged (10 min, 20817 *g*, 4°C), and 200 μl of the supernatant were used undiluted for HPLC. The autosampler kept the samples at 4°C until measuring. The chromatograph (Shimadzu, USA) had a Hi-Plex H column (300 mm length, 7.7 mm diameter, Agilent Technologies, UK) running at 60°C. The injection volume was 20 μl and the running time 40 min. As eluent 5 mM H_2_SO_4_ was used with a flow rate of 0.6 ml min^−1^. The same substances as above were detected with a reflexion index detector and analysed with the software LabSolutions (Shimadzu, Japan). The pH of the culture liquid was analysed from a 100 μl subsamples taken for HPLC analysis.

#### Cell sampling

Before a culture was transferred into fresh medium, a sample for molecular analysis was taken. The pellet obtained during the first centrifugation step of the liquid sample taken for HPLC analysis was re-suspended in 1 mM PBS buffer (pH 7, 8.00 g l^−1^ NaCl, 0.20 g l^−1^ KCl, 0.024 g l^−1^ KH_2_PO_4_, 0.144 g l^−1^ Na_2_HPO_4_) and centrifuged (10 min, 20817 *g*, room temperature). The supernatant was discarded and the pellet stored at −20°C.

### DNA isolation and PCR

Deoxyribonucleic acid was isolated from the original sediment samples (200 mg) and the enrichment cultures (cell pellet from 900 μl liquid culture, see above) using FAST DNA Spin for Soil-Kit (MP Biomedicals, USA) according to the provided protocol with the following modifications: two subsequent bead beating steps were applied at level 6 for 30 s, and the DNA was eluted in 70 μl nuclease-free distilled water. Deoxyribonucleic acid quantity and quality were determined by gel electrophoresis and photometric measurement using NanoDrop ND-1000 UV/Vis spectral photometer (PeqLab, Germany).

Colonies on agar plates were picked in the anaerobic chamber with sterile tooth picks and transferred into 96-well plates filled with 50 μl sterile, nuclease-free water into each well. In order to disrupt the cells, three cycles of heat shock (3 min, 95°C) and freezing (20 min, −20°C) were applied. The samples were centrifuged (20 min, 2200 *g*, room temperature), and supernatant was used for bacterial 16S rRNA gene amplification.

Bacterial 16S rRNA gene fragments were amplified using the primers 27F and 1492R (Lane, 1991), while the forward primer mlas and reverse primer mcrA-rev were used for the amplification of the mcrA gene as described by Steinberg and Regan (2008). The PCR reaction mixture contained 200 mM of each deoxynucleoside triphosphate, 0.5 U of HotStarTaq Plus DNA Polymerase (Qiagen, Germany), 1X Taq buffer with 2 μl of each primer (5.0 pmol) and 1 μl of genomic DNA template (100-fold diluted in case of original sediment samples) in a total volume of 20 μl. In case of PCR for subsequent T-RFLP analysis, the 27F primer (or mcrA-rev) was 5’-labeled with the phosphoramidite fluorochrome 6-carboxyfluorescein. The thermocycling program was as follows: initial denaturation at 95°C for 5 min followed by 30 cycles (25 cycles in case of DNA isolated from colonies) of denaturation at 94°C for 45 s, primer annealing at 51°C for 30 s, chain extension at 72°C for 2 min and a final extension at 72°C for 30 min. The mcrA gene was amplified as described earlier (Steinberg and Regan, 2008). Polymerase chain reaction products were separated on a 1.5% agarose gel, stained with ethidium bromide and visualized with UV excitation. Polymerase chain reaction products were purified using the QIAquick PCR Purification Kit (Qiagen, Germany). Polymerase chain reaction products of colonies were purified using SureClean Kit (Bioline GmbH, Germany).

### T-RFLP analysis

Purified PCR products (10–20 ng per community sample, 1 ng per sample for clone PCR products) were digested over night at 37°C in a 10 μl reaction volume with 5 U of either the restriction endonuclease HaeIII or MspI (New England Biolabs, Germany). Samples were ethanol precipitated, and dried DNA pellets were re-suspended in 10 μl HiDi formamide containing 1.5% (v/v) MapMarker 1000 (Bioventures, USA) or GeneScan-500 ROX standard (Applied Biosystems, Germany) in case of *mcrA/mrtA* products. Fluorescently labeled terminal restriction fragments (T-RFs) were separated on an ABI PRISM 3130xl Genetic Analyser (Applied Biosystems, Germany) with POP-7 polymer. The lengths of the terminal fragments were determined using GeneMapper V3.7 software (Applied Biosystems, Germany) and fluorescent data for the range of 50–1000 bp was exported (50–500 in case of *mcrA/mrtA*) to R script, and peak areas were normalized and noise filtering was applied (σ = 5) according to Abdo and colleagues (2006). A multivariate cluster analysis with the Bray–Curtis dissimilarity index was performed in program past (Hammer *et al*., 2001). In addition to the presence and absence of certain T-RFLP peaks, the calculations take the relative abundance ratios into consideration.

### Cloning and sequence analysis

Cloning of purified non-labelled PCR products was carried out with QIAGEN PCR Cloning Kit (Qiagen, Germany) according to the manufacturer’s protocol. Vector-specific M13 primers were used to re-amplify the inserted 16S rRNA amplicons. Before sequencing, the PCR products were screened by T-RFLP as described earlier. The mlas and mcrA-rev primers were used for the re-amplification of the inserts from selected *mcrA/mrtA* clones as described before (Nikolausz *et al*., 2013). Polymerase chain reaction products were screened by T-RFLP analysis to select clones with inserts matching the predominant peaks of the community T-RFLP patterns. A simplified protocol was used for the T-RFLP screening of the clones by omitting the purification of PCR products and ethanol precipitation of the restriction enzyme digestion. Partial sequencing of SAP-ExoI enzyme-treated PCR products was performed with 519r for 16S rRNA gene amplicon and mcrA-rev primer using the Big Dye Terminator Ready Reaction Cycle Sequencing Kit 1.1 and an ABI PRISM 3130xl Genetic Analyzer (Applied Biosystems, Germany). Almost full sequencing of the 16S rRNA genes of the selected strains was achieved by assembling sequence information from sequences obtained with primers 27F, 357F, 530F, 519R, 1100R, 1492R (Lane, 1991) using Sequencher version 4.8 sequence analysis software (Gene Codes Corporation, USA) The blastn and blastx algorithms (Altschul *et al*., 1997) were used to search for similar sequences in the public databases. The DNA sequences obtained in this study were deposited in EMBL public database under the accession numbers LK391546-LK391596 (16S rRNA gene) and LK391597-LK391611 (*mcrA/mrtA* genes). Additional phylogenetic analyses including phylogenetic tree generation were conducted using mega4 software (Tamura *et al*., 2007).

## Supporting Information

**Fig. S1.** (A) Normalized semi-quantitative cumulative gas production in relation to the medium volume, (B) pH values, (C) relative CH_4_ concentration and (D) relative CO_2_ concentration at the end of incubation of the first enrichment (En) and the subsequent transfers of the cultures from Lake Velencei (V37°C, ●) and Lake Szarvas (S37°C, ) incubated at 37°C and of the culture from Lake Szarvas incubated at 55°C (S55°C, ▲). The last day of measurement of the different parameters are listed in Table S2. The third transfer of S55°C was not sampled. The fourth transfer of S55°C was performed in parallel with the fifth transfer of V37°C and S37°C leading to different transfer numbers for transfers performed at the same time (see also Table S1). **Fig. S2.** Phylogenetic affiliation of *mcrA/mrtA* clone sequences obtained from the fifth and sixth transfer of the cultures S37°C and V37°C (enriched from sediment of the Lake Szarvas and Velencei, respectively, and incubated at 37°C) and from the fourth transfer of the culture S55°C (enriched from sediment of Lake Szarvas and incubated at 55°C) based on currently available sequences of related cultivable methanogens. The evolutionary history was inferred using the neighbour-joining method (substitution type: nucleotide). Bootstrap values expressed as percentages of 1000 replications are shown next to the branches. **Fig. S3.** Horizontal U-tube used to determine the produced gas volume in the culture bottles semi-quantitatively. The U-tube was filled with a solution of 200 g l^−1^ NaCl and 5 g l^−1^ citric acids (Rozzi and Remigi, 2004). Photo: Lisa Reither, 2013, UFZ. **Table S1.** Overview of the different enrichment cultures and their transfers. For each lake, three enrichment cultures were set up with sediment samples taken at different sites of the lakes. Enrichment cultures with sediment from the reed bed were incubated at 37°C and 55°C, cultures with sediment sampled outside the littoral zone were incubated at 37°C. Wheat straw of 10 mm length was used as lignocellulose source. x indicates the performed enrichments and transfers. **Table S2.** Last day of measurement of the gas volume (gasV), the pH of the medium and the composition of the gas phase (GC) of the cultures V37°C and S37°C (both were incubated at 37°C and were inoculated with sediment from Lake Velencei and Lake Szarvas, respectively) and of the culture S55°C (incubated at 55°C and inoculated with sediment from Lake Szarvas). **Table S3.** Relative gas concentration in the headspace of the sixth transfer (6.T) of the enrichment cultures from Lake Velencei (V37°C) and Lake Szarvas (S37°C) incubated at 37°C, of the fifth transfer (5.T) of the enrichment culture from Lake Szarvas incubated at 55°C (S55°C), and of the negative controls incubated at 37°C (NC37°C) and 55°C (NC55°C). The controls contained the same medium and amount of straw as the cultures. **Table S4.** Sequencing results of representative 16S rRNA gene clones and experimentally determined terminal restriction fragment (T-RF) sizes.
