# Supplementary material for: Characterization of wheat straw-degrading anaerobic alkali-tolerant mixed cultures from soda lake sediments by molecular and cultivation techniques
Source: Microb Biotechnol. 2015 Mar 4;8(5):801–14. doi: 10.1111/1751-7915.12272 (PMC4554468; doi:10.1111/1751-7915.12272)
Supplement: Supplementary file 1 [file mbt20008-0801-sd1.docx]

**Characterization of wheat straw-degrading anaerobic alkali-tolerant mixed cultures from soda lake sediments by molecular and cultivation techniques**

Katharina Porsch^1,2^, Balázs Wirth^3^, Erika M. Tóth^3^, Florian Schattenberg^1,2^, Marcell Nikolausz^1,2*^

^1^Department of Bioenergy, Helmholtz Centre for Environmental Research – UFZ, Leipzig, Germany

^2^Department of Environmental Microbiology, Helmholtz Centre for Environmental Research – UFZ, Leipzig, Germany

^3^Department of Microbiology, Eötvös Loránd University, Budapest, Hungary

* Corresponding author:

Mailing address: Helmholtz Centre for Environmental Research – UFZ, Department of Environmental Microbiology, Permoserstr. 15, 04318 Leipzig, Germany

Phone: +49 341 2434 566

Fax: +49 341 2434 133

E-mail: marcell.nikolausz@ufz.de

**Supporting Information**

**Table S1**

Overview of the different enrichment cultures and their transfers. For each lake three enrichment cultures were setup with sediment samples taken at different sites of the lakes. Enrichment cultures with sediment from the reed-bed were incubated at 37°C and 55°C, cultures with sediment sampled outside the littoral zone were incubated at 37°C. Wheat straw of 10 mm length was used as lignocellulose source. x indicates the performed enrichments and transfers.

|  | **Lake Szarvas** | | | **Lake Velencei** | | |
| --- | --- | --- | --- | --- | --- | --- |
| Sediment sample | Reed-bed | Reed-bed | Outside littoral zone | Reed-bed | Reed-bed | Outside littoral zone |
| Cultivation temp. [°C] | 55 | 37 | 37 | 55 | 37 | 37 |
| Abbreviation | S55°C | **-** | S37°C | **-** | **-** | V37°C |
| 1. Enrichment | x | x | x | x | x | x |
| 1. Transfer | x | x | x | x | x | x |
| 2. Transfer | x | x | x | x | x | x |
| 3. Transfer | x^a^ | **-** | x | **-** | x^a^ | x |
| 4. Transfer | **-** | **-** | x | **-** | **-** | x |
| 5. Transfer | x^b^ (4. Transfer) | **-** | x | **-** | **-** | x |
| 6. – 9. Transfer | x  (5. – 8. Transfer) | **-** | x | **-** | **-** | x |

a – Culture was not sampled and analyzed

b – The 4^th^ transfer of this culture was performed in parallel with the 5^th^ transfer of the cultures S37°C and V37°C.

**
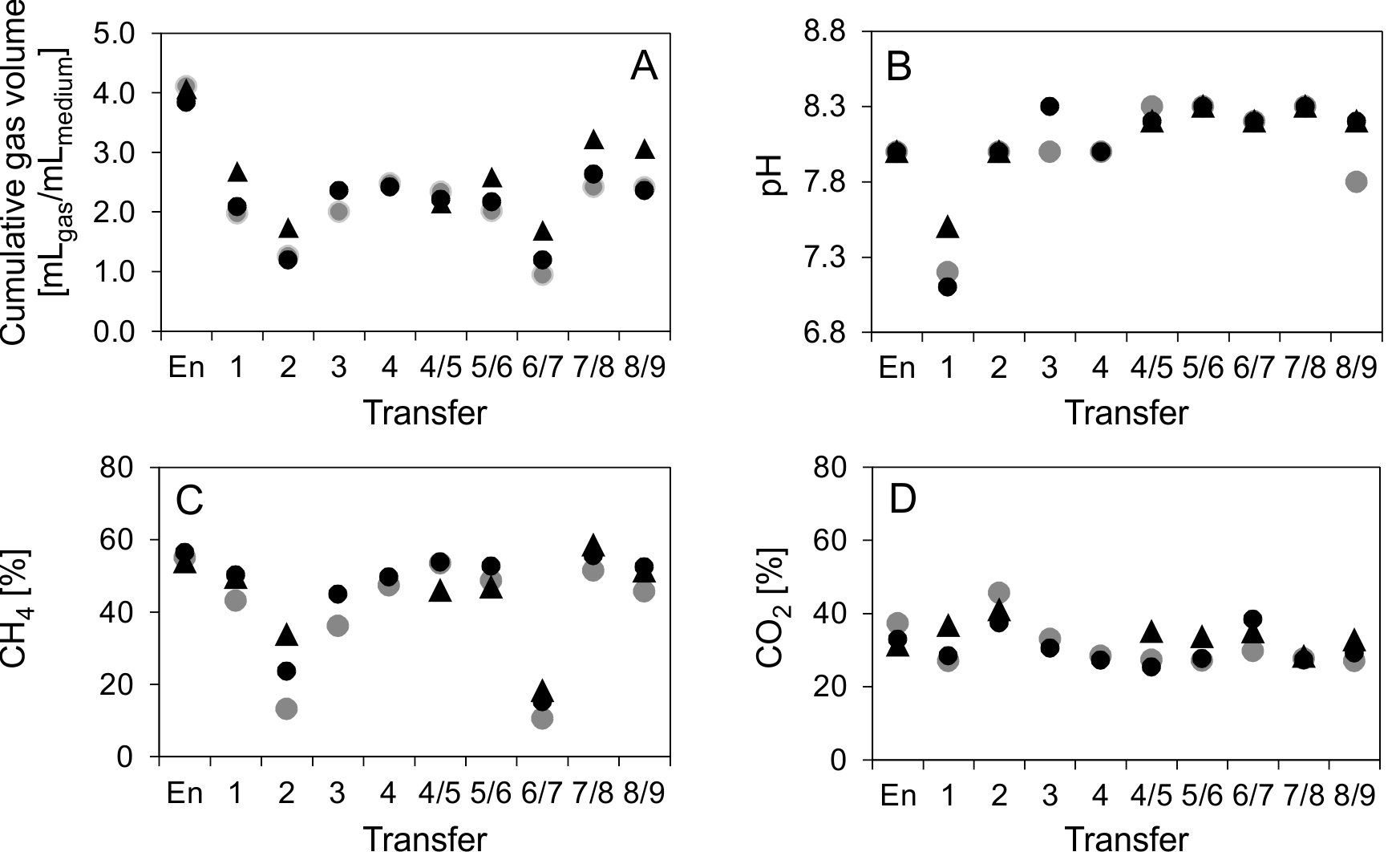
**

**Fig. S1.** A) Normalized semiquantative cumulative gas production in relation to the medium volume, B) pH values, C) relative CH_4_ concentration, and D) relative CO_2_ concentration at the end of incubation of the first enrichment (En) and the subsequent transfers of the cultures from Lake Velencei (V37°C, ●) and Lake Szarvas (S37°C, ●) incubated at 37°C and of the culture from Lake Szarvas incubated at 55°C (S55°C, ▲). The last day of measurement of the different parameters are listed in Table S2. The 3^rd^ transfer of S55°C was not sampled. The 4^th^ transfer of S55°C was performed in parallel with the 5^th^ transfer of V37°C and S37°C leading to different transfer numbers for transfers performed at the same time (see also Table S1).

**Table S2**

Last day of measurement of the gas volume (gasV), the pH of the medium and the composition of the gas phase (GC) of the cultures V37°C and S37°C (both were incubated at 37°C and were inoculated with sediment from Lake Velencei and Lake Szarvas, respectively) and of the culture S55°C (incubated at 55°C and inoculated with sediment from Lake Szarvas).

| **Transfer** | **Culture V37°C** | **Culture S37°C** | **Transfer** | **Culture S55°C** |
| --- | --- | --- | --- | --- |
|  | gasV / pH / GC | gasV / pH / GC |  | gasV / pH / GC |
| Enrichment | 61 / 61 / 62 | 61 / 61 / 62 | Enrichment | 61 / 61 / 62 |
| 1st | 34 / 27 / 29 | 34 / 27 / 29 | 1st | 34 / 27 / 29 |
| 2nd | 38 / 38 / 40 | 38 / 38 / 40 | 2nd | 38 / 38 / 40 |
| 3rd | 38 / 31 / 26 | 38 / 31 / 26 | 3rd | Not sampled |
| 4th | 31 / 31 / 26 | 31 / 31 / 26 | - |  |
| 5th | 28 / 28 / 28 | 28 / 28 / 28 | 4th | 35 / 35 / 35 |
| 6th | 30 / 30 / 30 | 30 / 30 / 30 | 5th | 30 / 30 / 30 |
| 7th | 25 / 25 / 26 | 25 / 25 / 26 | 6th | 25 / 25 / 26 |
| 8th | 42 / 42 / 42 | 42 / 42 / 42 | 7th | 42 / 42 / 42 |
| 9th | 30 / 30 / 30 | 30 / 30 / 30 | 8th | 30 / 30 / 30 |

**Table S3**

Relative gas concentration in the headspace of the 6^th^ transfer (6.T) of the enrichment cultures from Lake Velencei (V37°C) and Lake Szarvas (S37°C) incubated at 37°C, of the 5^th^ transfer (5.T) of the enrichment culture from Lake Szarvas incubated at 55°C (S55°C), and of the negative controls incubated at 37°C (NC37°C) and 55°C (NC55°C). The controls contained the same medium and amount of straw as the cultures.

| **Culture** | **Time [d]** | **N_2_ [%]** | **H_2_ [%]** | **CO_2_ [%]** | **CH_4_ [%]** |
| --- | --- | --- | --- | --- | --- |
| V37°C 6.T | 0 | 98 | 2 | 0 | 0 |
|  | 9 | 45 | 0 | 30 | 25 |
|  | 16 | 30 | 0 | 29 | 41 |
|  | 26 | 25 | 0 | 28 | 47 |
|  | 30 | 24 | 0 | 27 | 49 |
| S37°C 6.T | 0 | 98 | 2 | 0 | 0 |
|  | 9 | 45 | 0 | 35 | 20 |
|  | 16 | 28 | 0 | 30 | 42 |
|  | 26 | 24 | 0 | 27 | 49 |
|  | 30 | 20 | 0 | 28 | 53 |
| S55°C 5.T | 0 | 98 | 2 | 0 | 0 |
|  | 9 | 37 | 0 | 42 | 21 |
|  | 16 | 28 | 0 | 40 | 32 |
|  | 26 | 21 | 0 | 35 | 44 |
|  | 30 | 20 | 0 | 34 | 47 |
| NC37°C | 0 | 98 | 2 | 0 | 0 |
|  | 9 | 94 | 2 | 4 | 0 |
|  | 16 | 93 | 2 | 5 | 0 |
|  | 26 | 90 | 3 | 7 | 0 |
|  | 30 | 89 | 5 | 6 | 0 |
| NC55°C | 0 | 98 | 2 | 0 | 0 |
|  | 9 | 93 | 2 | 5 | 0 |
|  | 16 | 92 | 1 | 7 | 0 |
|  | 26 | 92 | 1 | 7 | 0 |
|  | 30 | 91 | 1 | 7 | 0 |

**Table S4**

Sequencing results of representative 16S rRNA gene clones and experimentally determined terminal restriction fragment (T-RF) sizes.

| **Clone ID**  **(accession number)** | **Classification*** | **Highest BLAST hit (Acc. No.) / Sequence identity**  **Closest cultured relative (Acc. No.) / Sequence identity** | TRF size (*Msp*I) |
| --- | --- | --- | --- |
| **S37** |  |  |  |
| S37_1.4  (LK391572) | Flavobacteriaceae | Uncultured *Clostridium* sp. clone 30-NS40 from biofilm on electrode material in a microbial fuel cell (JQ724353) 100%  *Eubacterium* sp. F1 from the rumen of cattle (EU281854) 86% | 79 |
| S37_1.5  (same as S37_1.4) | Flavobacteriaceae | Uncultured *Clostridium* sp. clone 30-NS40 from biofilm on electrode material in a microbial fuel cell (JQ724353) 100%  *Eubacterium* sp. F1 from the rumen of cattle (EU281854) 86% | 79 |
| S37_5.1  (same as S37_1.4) | Flavobacteriaceae | Uncultured *Clostridium* sp. clone 30-NS40 from biofilm on electrode material in a microbial fuel cell (JQ724353) 100%  *Eubacterium* sp. F1 from the rumen of cattle (EU281854) 86% | 79 |
| S37_5.3  (same as S37_1.4) | Flavobacteriaceae | Uncultured *Clostridium* sp. clone 30-NS40 from biofilm on electrode material in a microbial fuel cell (JQ724353) 100%  *Eubacterium* sp. F1 from the rumen of cattle (EU281854) 86% | 79 |
| (LK391573) | Marinilabiaceae | Uncultured bacterium clone 01f03 from ASBR reactor treating swine waste (GQ135836) 99%  *Alkalitalea saponilacus* strain SC/BZ-SP2 (HQ191474) 95% | 84 |
| S37_7.5  (LK391574) | *Paludibacter* | Uncultured bacterium clone MEB2_931 from Microbial Electrolysis Cell cathode biofilm (JQ987960) 100%  *Paludibacter propionicigenes* WB4 (CP002345) 92% | 86 |
| S37_4.5  (LK391575) | Spirochaetaceae | Uncultured *Spirochaeta* sp. clone wn13 from activated sludge (JQ012260) 99%  *Sphaerochaeta* sp. RCcp2 strains isolated from dechlorinating enrichment cultures (DQ833401) 92% | 117 |
| S37_6.6  (same as S37_4.5) | Spirochaetaceae | Uncultured *Spirochaeta* sp. clone wn13 from activated sludge (JQ012260) 99%  *Sphaerochaeta* sp. RCcp2 strains isolated from dechlorinating enrichment cultures (DQ833401) 92% | 117 |
| S37_2.6  (LK391576) | Unclassified Bacteria | *Sphaerochaeta* sp. GLS2 from (JN944166) 99%  Spirochaeta sp. Buddy isolated from marine hot spring (CP002541) 99% | 122 |
| S37_6.5  (LK391577) | Sphingobacteriales  Cytophagaceae | *Meniscus glaucopis* (92%)  Uncultured bacterium clone NK-M4 (water flooding oil reservoirs) (92%) | 199 |
| S37_7.6  (LK391578) | Sphingobacteriales  Cytophagaceae | *Meniscus glaucopis* (91%)  Uncultured bacterium clone SLE39H from anaerobic digester treating feedstock (90%) | 199 |
| S37_8.3  (LK391579) | unclassified_Spirochaetaceae | Spirochaetes bacterium SA-8 (89%)  Uncultured bacterium clone BP_U4C_3a09 from lab-scale upflow anaerobic bioreactors (99%) | 205 |
| S37_8.6  (LK391580) | unclassified_Spirochaetaceae | Spirochaetes bacterium SA-8 (89%)  Uncultured bacterium clone BP_U4C_3a09 from lab-scale upflow anaerobic bioreactors (99%) | 205 |
| S37_4.6  (LK391581) | Spirochaetaceae | *Spirochaeta zuelzerae* (96%)  Uncultured bacterium clone J2_3_125 from full-scale anaerobic digesters (99%) | 279 |
| S37_8.4  (LK391582) | Spirochaetaceae | *Spirochaeta zuelzerae* (96%)  Uncultured bacterium clone J2_3_125 from full-scale anaerobic digesters (99%) | 279 |
| **S55** |  |  |  |
| S55_2.6  (LK391583) | Clostridiales | *Desulfotomaculum* sp. TGB60-1 (85%)  Uncultured bacterium clone B55_F_B_C06 from thermophilic anaerobic solid waste digestor (98%) | 1.22 |
| S55_5.6  (LK391584) | Clostridiales | *Desulfotomaculum* sp. TGB60-1 (85%)  Uncultured bacterium clone B55_F_B_C06 from thermophilic anaerobic solid waste digestor (98%) | 1.34 |
| S55_3.5  (LK391585) | unclassified Clostridia | *Moorella thermoacetica* (88%)  Uncultured bacterium partial 16S rRNA gene, clone 3wk_2LB17 (biowaste sludge reactor) | 1478 |
| S55_8.6  (LK391586) | unclassified Clostridiales | *Moorella thermoacetica* (86%)  Firmicutes bacterium enrichment culture clone WSC-21 (lignocellulose) | 148 |
| S55_8.7  (LK391587) | *Clostridium* (III) | *Clostridium stercorarium* (90%) (thermophilic)  Uncultured bacterium clone ATB-AR-23636 from a percolated hydrolysis reactor with rye silage (99%) | 165 |
| S55_3.8  (LK391588) | *Clostridium* (III) | *Clostridium straminisolvens* (93%) (moderately thermophilic)  Uncultured bacterium clone ATB-AR-23660 from a percolated hydrolysis reactor with rye silage | 214 |
| S55_6.8  (LK391589) | Lachnospiraceae | *Defluviitalea saccharophila* (87%) (thermophilic bacterium)  Uncultured bacterium clone ATB-AR-23648 from a percolated hydrolysis reactor with rye silage (99%) | 214 |
| S55_7.6  (LK391590) | Lachnospiraceae | *Defluviitalea saccharophila* (87%) (thermophilic bacterium)  Uncultured bacterium clone ATB-AR-23648 from a percolated hydrolysis reactor with rye silage (99%) | 301 |
| S55_8.5  (same as S55_6.8) | Lachnospiraceae | *Defluviitalea saccharophila* (87%)  Uncultured bacterium clone ATB-AR-23648 from a percolated hydrolysis reactor with rye silage (99%) | 301 |
| S55_2.7  (LK391591) | *Lutispora* (Gracilibacteraceae) | *Lutispora thermophila* (96%)  Uncultured bacterium clone T1_3_1968 from full-scale anaerobic digesters (98%) | 301 |
| S55_4.7 | *Lutispora* (Gracilibacteraceae) | *Lutispora thermophila* (99%)  Uncultured bacterium clone J2_2_2681 from full-scale anaerobic digesters (100%) | 304 |
| **V37** |  |  |  |
| V37_7.7  (LK391592) | unclassified_Clostridiales | *Moorella glycerini* (95%) (homoacetogenic thermophilic) | 148 |
| V37_1.7  (LK391593) | unclassified_Clostridiales | *Caloranaerobacter azorensis* (90%) | 148 |
| V37_2.6  (LK391594) | Sphingobacteriales | Bacteroidetes bacterium 4F6B isolated from rice field soil in Japan (98%)  Uncultured bacterium clone BP_U1C_1f07 from lab-scale upflow anaerobic bioreactors (99%) | 540 |
| V37_3.7  (LK391595) | Sphingobacteriales | Bacteroidetes bacterium 4F6B isolated from rice field soil in Japan (98%)  Uncultured bacterium clone BP_U1C_1f07 from lab-scale upflow anaerobic bioreactors (100%) | 540 |
| V37_5.7  (LK391596) | Sphingobacteriales | Bacteroidetes bacterium 4F6B isolated from rice field soil in Japan (98%)  Uncultured bacterium clone BP_U1C_1f07 from lab-scale upflow anaerobic bioreactors (100%) | 540 |

* 50% confidence threshold was applied in the Classifier of Ribosomal Database Project (RDP, release 10)


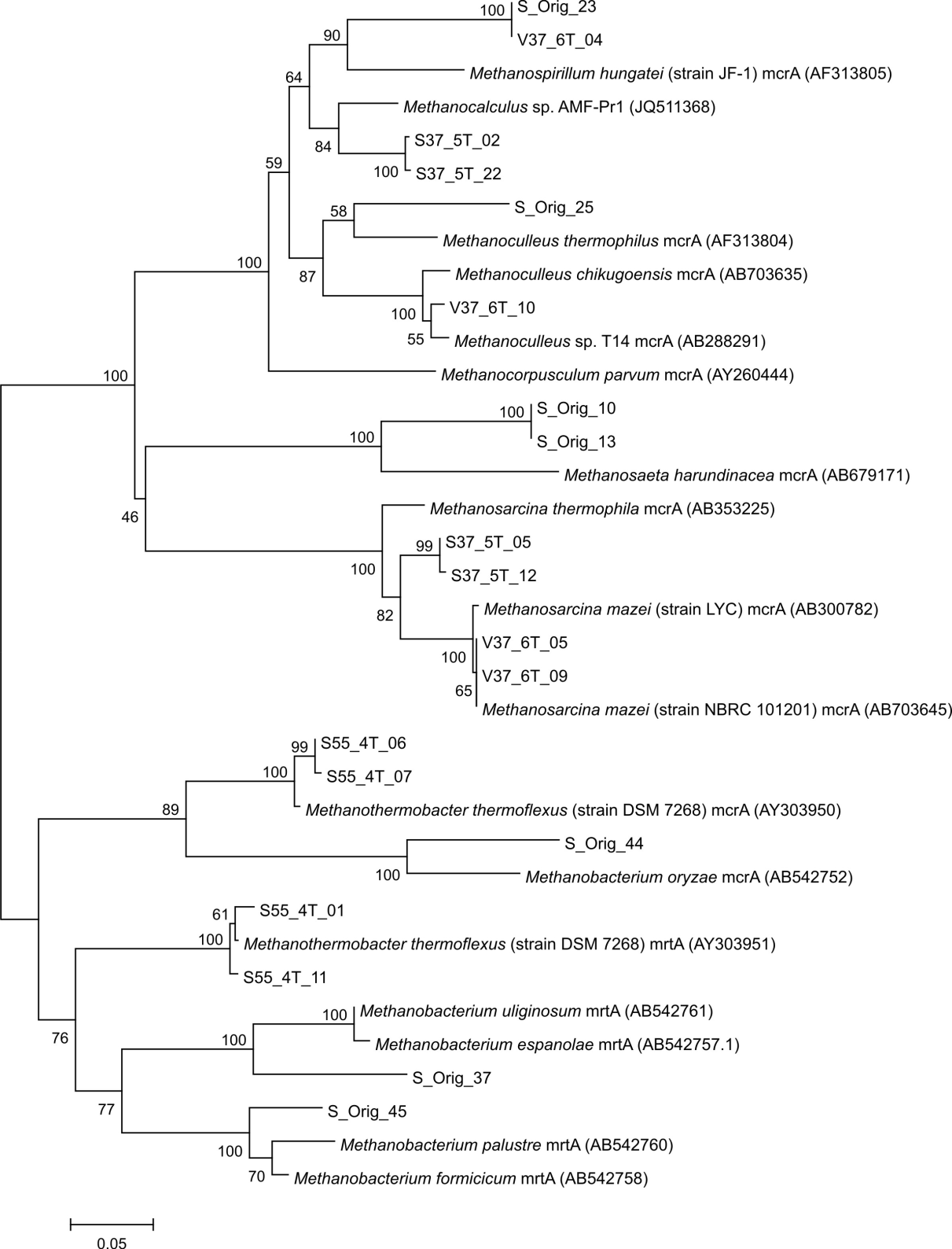


**Fig. S2.** Phylogenetic affiliation of *mcrA/mrtA* clone sequences obtained from the 5^th^ and 6^th^ transfer of the cultures S37°C and V37°C (enriched from sediment of the Lake Szarvas and Velencei, respectively, and incubated at 37°C) and from the 4^th^ transfer of the culture S55°C (enriched from sediment of Lake Szarvas and incubated at 55°C) based on currently available sequences of related cultivable methanogens. The evolutionary history was inferred using the neighbor-joining method (substitution type: nucleotide). Bootstrap values expressed as percentages of 1000 replications are shown next to the branches.


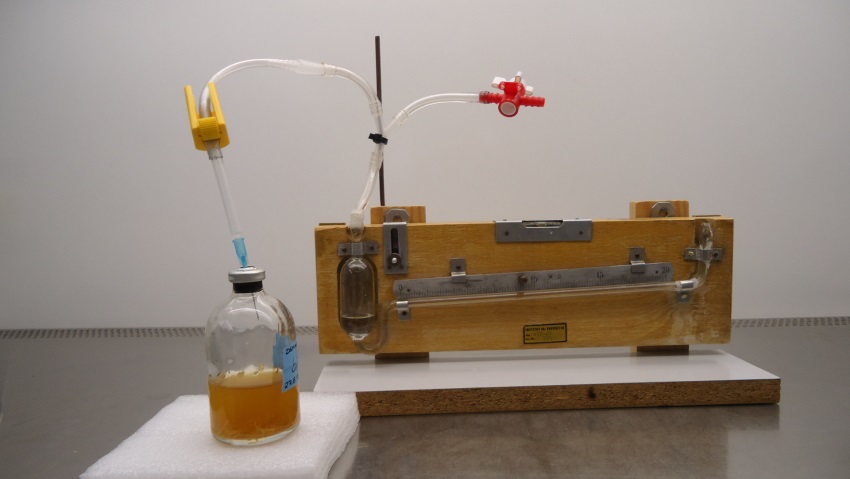


**Fig. S3.** Horizontal U-tube used to determine the produced gas volume in the culture bottles semi-quantitatively. The U-tube was filled with a solution of 200 g/L NaCl and 5 g/L citric acids ([Rozzi and Remigi, 2004](#_ENREF_27)). Photo: Lisa Reither, 2013, UFZ.
